# Impact of Cervical Spine Rehabilitation on Temporomandibular Joint Functioning in Patients with Idiopathic Neck Pain

**DOI:** 10.1155/2021/6886373

**Published:** 2021-10-07

**Authors:** Łukasz Oleksy, Renata Kielnar, Anna Mika, Agnieszka Jankowicz-Szymańska, Dorota Bylina, Jarosław Sołtan, Błażej Pruszczyński, Artur Stolarczyk, Aleksandra Królikowska

**Affiliations:** ^1^Orthopaedic and Rehabilitation Department, Medical University of Warsaw, Poland; ^2^Oleksy Medical & Sports Sciences, Poland; ^3^Institute of Health Sciences, Medical College of Rzeszów University, Poland; ^4^Institute of Clinical Rehabilitation, University of Physical Education in Kraków, Poland; ^5^Institute of Health Sciences, State Higher Vocational School in Tarnów, Poland; ^6^Chair of Natural Sciences, Józef Pilsudski University of Physical Education in Warsaw, Faculty of Physical Education in Biała Podlaska, Poland; ^7^Foreign Languages Department, The Josef Pilsudski University of Physical Education in Warsaw, Faculty of Physical Education in Biała Podlaska, Poland; ^8^Department of Orthopaedics and Paediatric Orthopaedics Medical University of Łódź, Poland; ^9^Ergonomics and Biomedical Monitoring Laboratory, Departament of Physiotherapy, Faculty of Health Sciences, Wroclaw Medical University, Poland

## Abstract

**Objective:**

The aim of this study was to assess the effectiveness of a 3-week rehabilitation programme focusing only on the cervical region, pain intensity, range of motion in the cervical spine, head posture, and temporomandibular joint (TMJ) functioning in subjects with idiopathic neck pain who did not report TMJ pain.

**Design:**

A parallel group trial with follow-up.

**Methods:**

The study included 60 participants divided into 2 groups: experimental: *n* = 25, 27-57 years old, experiencing idiopathic neck pain and who underwent a 3-week rehabilitation programme, and the control, *n* = 35, 27-47 years, who were cervical pain-free. At baseline and after 3 weeks of treatment in the experimental group and with a 3-week time interval in the control group, pain intensity, head posture in the sagittal plane, range of motion in the cervical spine, and TMJ functioning were evaluated.

**Results:**

After 3 weeks of rehabilitation, there was a significant decrease in pain intensity, improved range of motion of the cervical spine and head posture, and improved clinical condition of TMJ in participants with idiopathic neck pain who did not report TMJ pain.

**Conclusion:**

The study suggested that idiopathic neck pain is associated with limited range of motion in the cervical spine, incorrect head posture, and TMJ dysfunction. Our data suggests that therapy focusing only on the cervical region may improve the clinical condition of the TMJ in subjects with idiopathic neck pain who do not report TMJ pain. These observations could be helpful in physiotherapeutic treatment of neck and craniofacial area dysfunctions. This trial is registered with ISRCTN Registry ISRCTN14511735.

## 1. Introduction

Approximately 70% of the general population experience neck pain or decreased quality of everyday life due to cervical spine dysfunctions [1]. This constitutes an essential health issue, and the magnitude of its socioeconomic outcomes is secondary only to low back pain [2, 3]. In most cases, it is difficult to identify the cause of neck pain unambiguously, which is a substantial obstacle when customising congruent therapy and preventing further recurrences [4, 5]. Chronic idiopathic neck pain is defined as neck pain lasting more than 3 months, without the presence of trauma, cervical hernias with clinical symptoms, or radiculopathy. On the basis of its etiology, it was divided into specific neck pain, trauma-induced neck pain, and idiopathic (nontraumatic) neck pain [6]. The complicated anatomical structure of the cervical spine, its complex biomechanical function, close proximity of nervous system structures, and symptom inhomogeneity are a challenge for clinicians and researchers dealing with diagnostics and treatment of neck pain.

It has been reported that the majority of the general population experience signs of temporomandibular joint (TMJ) dysfunction [7–10]. Some authors have reported a relationship between craniofacial and neck pain, including biomechanical, neuroanatomical, and neurophysiological aspects [11, 12]. Close anatomical connection of the cervical spine to the masticatory system and frequent comorbidity of the neck and temporomandibular joint dysfunction (TMD) suggest the need to study the relationship between these areas [7, 13–15]. Incorrect tension of the masticatory muscles was found to be associated with head posture and was suggested as one of the causes of dysfunctions in cervical paravertebral muscles [16, 17]. The possible explanation could be the neurophysiologic connections between the cervical spine and temporomandibular area, such as the convergence of trigeminal and upper cervical afferent inputs in the trigeminocervical nucleus [12]. As was described by some authors, different afferent nerve fibers converge onto the trigeminal nuclei, causing an overlap between cervical and trigeminal afferents in the trigeminal nucleus caudalis [11].

There are some existing reports in which it is stated that long-term upper cervical dysfunction may influence the functioning of the temporomandibular region and vice versa [18], but most often, only the relationship between these areas is described [18–20], without any proposed therapy. Although the association of cervical spine disorders and TMD has been studied by different authors, it is far from being exhaustively explained. Moreover, some authors have evaluated the effects of therapy on subjects with TMD only [21–25] or on those reporting pain in both areas [26]. Previous studies also evaluated the effects of the therapy including only the orofacial region [18, 24, 25], both (TMJ and neck) [11, 14, 26], or only the neck but including patients with TMJ pain [23, 26]. In none of the studies, the effect of neck therapy on changes in TMJ has been assessed in patients with neck pain alone, without concurrent TMJ. Based on the neurophysiological and biomechanical relationships between TMJ and the neck, it may be hypothesised that patients with idiopathic neck pain may also experience some TMJ dysfunctions, even those asymptomatic.

In this study, we aimed to analyse the effectiveness of a 3-week rehabilitation programme on pain intensity, range of motion in the cervical spine, head posture, and temporomandibular joint functioning in subjects with idiopathic neck pain who did not report TMJ pain. The novelty of this study is comprehensive analysis of the impact of neck-only therapy, without direct intervention in the craniofacial area on clinical condition of the TMJs. This is the first study in which it was aimed at checking whether the therapy focusing only on the cervical region in patients who did not report TMJ pain, but neck pain alone, has any real impact on the structures located in the craniofacial area.

## 2. Material and Methods

### 2.1. Participants

**Figure 1:**
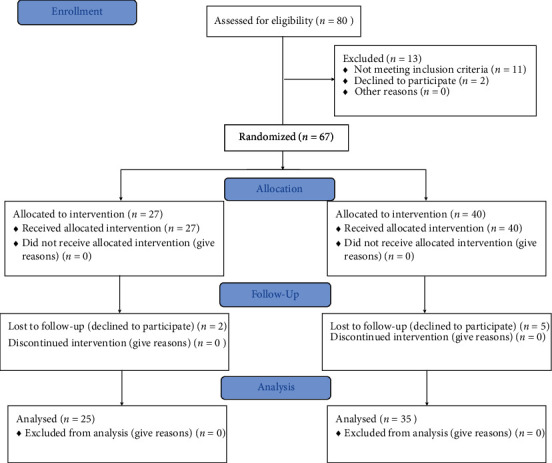
Consort flow diagram.

The study included a group of 60 participants divided into 2 groups (Figure 1). Subjects from both groups were evaluated with manual tests by a specialist physician who confirmed the diagnosis of idiopathic cervical pain and qualified them to the experimental or control groups. Idiopathic neck pain was diagnosed when the following symptoms were present: neck and shoulder girdle muscle stiffness, headaches, vertigo, paresthesias, referred pain, pain with palpation of the trigger point, and limited range of motion. Participants with chronic idiopathic neck pain reported the following at the time of evaluation: chronic, persistent, deep aching neck pain in soft tissues at least 4/10 on a VAS scale, experience of pain for 12 weeks or longer, and pain associated with activities and relieved with rest. Neck pain was considered idiopathic when the onset was spontaneous and the cause was unknown [6]. All subjects from the experimental group were patients of the Rehabilitation Clinic who received ambulatory treatment. They were recruited from patients admitted to the Clinic. Age-matched control subjects were recruited from the local community.

Group 1 (experimental) (*n* = 25, 27-57 years old (38.5 ± 8.52)) included those experiencing chronic idiopathic neck pain and who underwent a 3-week rehabilitation programme.

Exclusion criteria were

- cervical spine injury 3 months prior to the therapy
- regular use of painkillers or steroids without possibility of their withdrawal for the whole duration of the therapy
- radiographically diagnosed developmental or degenerative abnormalities of the cervical spine, such as spinal stenosis, subluxations, and spinal disc herniation
- orthodontic treatment (braces, aligners)
- removable dentures

Group 2 (control) (*n* = 35, aged 27-47 years (35.1 ± 5.65)) included those who were cervical pain-free, had no cervical spine or TMJ dysfunctions, and were not in the process of current orthodontic treatment. Subjects from the control group did not undergo any therapy.

All participants were informed in detail about the research protocol and gave their written informed consent to participate in the study. This study was approved by the Ethical Committee of Rzeszów University in Poland. All procedures were performed in accordance with the 1964 Declaration of Helsinki and its later amendments. This study was registered in the ISRCTN registry, Registration number: ID ISRCTN14511735.

### 2.2. Experimental Procedures

All measurements were performed twice, at baseline and after 3 weeks of treatment in the experimental group, and with a 3-week interval in the control group.

The main (primary) outcome measures were

1. pain intensity measured using a 10-point visual analogue scale
2. temporomandibular joint functioning measured using the Helkimo clinical dysfunction index
3. head posture and range of motion in the cervical spine—assessed with a measuring tape

#### 2.2.1. Pain Intensity

Pain intensity is evaluated with a 10-point visual analogue scale (VAS) [27].

#### 2.2.2. Head Posture in the Sagittal Plane

The distance between the jugular notch of the sternum and mental protuberance of the lower jaw was measured in centimetres twice, both times while seated, with habitual and neutral head posture (the subject was asked by the researcher to assume a position with the head within body axis avoiding protracted head position) [28]. Head posture was also evaluated visually—qualitatively differentiating between correct and forward head posture [29–32]. Measurements of the forward head position performed by the same physical therapist were highly reliable (ICC = 0.93). Good reliability (ICC = 0.83) was demonstrated when different physical therapists measured the forward head posture of the same patient [33].

#### 2.2.3. Range of Motion in the Cervical Spine

The distance in centimetres was measured with a tape measure in the following conditions: the reported ICC values ranged from 0.89 to 0.98 [34, 35].

- Flexion in upper segments of the cervical spine (measurement points: jugular notch in sternum-mental protuberance of the lower jaw)
- Flexion in lower segments of the cervical spine (measurement points: jugular notch in sternum-mental protuberance of the lower jaw)
- Extension in upper segments of the cervical spine (measurement points: jugular notch in sternum-mental protuberance of the lower jaw)
- Extension in lower segments of the cervical spine (measurement points: jugular notch in sternum-mental protuberance of the lower jaw)
- Cervical spine rotation to the right and left (measurement points: acromion-mental protuberance of the lower jaw) movement towards the acromion
- Cervical spine lateral flexion to the right and left (measurement points: acromion-mastoid process) movement towards the acromion

#### 2.2.4. Function of Temporomandibular Joints (TMJs)

The Helkimo clinical dysfunction index (Di) was used to measure the severity of temporomandibular joint dysfunctions. Subjects were qualified to 1 of the 4 groups: Di-0: lack of clinical dysfunction symptoms, Di-I: mild symptoms of dysfunction, Di-II: moderate dysfunction symptoms, and Di-III: severe dysfunction symptoms. The composite score of the Di value was also calculated [36, 37].

### 2.3. Therapeutic Interventions

The experimental group underwent a 3-week rehabilitation programme, individualized for each patient and comprising the following standard treatments for chronic pain of the musculoskeletal system [38–41]. The following techniques were performed.

#### 2.3.1. Manual Therapy (Soft Tissue Therapy of the Neck and the Shoulder Girdle: Trigger Point Therapy (Figure 2), Myofascial Release (Figure 3), and Classical Massage and Manual Cervical Traction (Figure 4))

The techniques were performed for 30 minutes each on the soft tissues which required relaxation depending on the patient's needs at a given time and progress of the whole therapy. At the end of manual soft tissue therapy, manual cervical traction was performed.

#### 2.3.2. Individual Exercises with a Therapist (Active Exercise (Figure 5), Body Posture Correction Exercises, and Respiratory Reeducation (Figure 6))

Active exercise included active correction of body posture, activation and training of the deep neck flexors, neck extensor and muscle stabilization, and rotation of the shoulder girdle. The exercises were performed in 3 series of 10 repetitions. Respirator reeducation included 3 series of 5-7 active breaths.

#### 2.3.3. Physical Therapy (Sollux Lamp)

Physical therapy (sollux lamp) is applied each time after soft tissue therapy on the shoulder girdle and cervical spine (15 minutes with a blue filter).

#### 2.3.4. Education on the Nature of the Dysfunction, Body Posture Correction Techniques, Sleep, and Everyday-Life Ergonomics

Rehabilitation was carried out 5 times a week, lasting about 2 hours per session. Participants from the control group did not undergo any therapy.

### 2.4. Statistical Analysis

Statistical analysis was carried out using the STATISTICA 12.0 software. The Shapiro-Wilk test was performed to assess the normality of variable distribution. Two-way ANOVA, with one main factor being the subjects (group: experimental vs. control) and the other main factor being the repeated measure (time: baseline vs. 3 weeks), was used to determine the significance of differences regarding the evaluated variables. After, the Tukey post hoc test was performed. The changes in VAS scale variables were assessed with the nonparametric Wilcoxon test. The effect size was calculated using Cohen's *d*. Differences were considered to be statistically significant at the level of *p* < 0.05. Paired *t*-test power analysis of rehabilitation influence determined that at least 25 subjects were required to obtain a power of 0.8 at a two-sided level of 0.05 with the effect size of *d* = 0.8.

## 3. Results

Patient characteristics are presented in Table 1.

### 3.1. Pain Intensity

**Figure 7:**
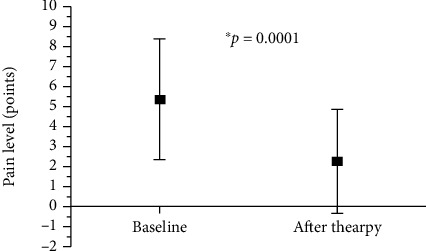
Pain intensity at baseline and after therapy in experimental group. ^∗^*p*: *p* value between baseline and posttherapy. Values are expressed as the mean ± SD.

All subjects in the experimental group reported cervical spine pain at the baseline. After the therapy, pain intensity decreased but was still present in 12 subjects, whereas it completely dissipated in 13 subjects. Cervical pain intensity significantly decreased after the applied therapy (Figure 7). None of the participants from the control group reported cervical pain at baseline or after the 3-week period.

### 3.2. Head Posture in the Sagittal Plane

At baseline, correct head posture was found in 6 participants from the experimental group (24%) and in 28 participants from the control group (80%). The rest of the participants, 19 in the experimental group (76%) and 7 from the control group (20%), demonstrated forward head posture. After 3 weeks of therapy, the number of participants with correct head posture increased to 18 (72%) in the experimental group. In the remaining 7 people from this group (28%), forward head posture was still observed. There were no changes in head posture in the control group (*p* > 0.05).

After the therapy in the experimental group, we observed a significantly lower distance between the jugular notch of the sternum and mental protuberance of the lower jaw in habitual as well as neutral head posture (Table 2). There were no significant changes in the control group (*p* > 0.05) (Table 2).

### 3.3. Range of Motion in Cervical Spine

A significant increase was noted in ranges of extension in the upper and the lower cervical spine, right side rotation, and right and left side lateral flexion after the applied therapy compared to baseline in the experimental group (Table 3). There were no significant changes in the control group (*p* > 0.05) (Table 3).

### 3.4. Function of Temporomandibular Joints (TMJs)

The Helkimo clinical dysfunction index (Di) demonstrated a greater occurrence of severe dysfunctions in the experimental compared to the control group. After the 3-week therapy, the number of participants with severe dysfunctions (Di-III) decreased by about two-thirds. There were no changes in the dysfunction index (Di) for the control group (*p* > 0.05).

At baseline, the dysfunction index (Di) was significantly greater in the experimental group (10.0 ± 4.84) compared to the control (6.0 ± 4.03) (*p* = 0.006). In the experimental group, the dysfunction index decreased significantly after therapy (5.6 ± 4.28) (*p* = 0.0001), reaching a value similar to that obtained in the control group (6.08 ± 3.89). There were no changes in the dysfunction index concerning the control group (*p* > 0.05).

## 4. Discussion

The most important observation from this study is that 3 weeks of rehabilitation significantly decreased pain intensity and improved range of motion of the cervical spine and head posture as well as the clinical condition of temporomandibular joints in participants with idiopathic neck pain who did not report TMJ pain. The effectiveness of the therapy, which focused only on the cervical region, has demonstrated impact on the structures located in the craniofacial area. In this study, it was shown how close the functional relationship between neck and temporomandibular joints is and, thus, how dysfunctions in each of these areas affect each other, even in patients without TMJ pain.

To date, some authors have reported the effectiveness of the rehabilitation in subjects with TMJ pain alone [18, 23, 24] or in those reporting pain in both areas [26]. There is a lack of studies, which included patients with neck pain alone without concurrent TMJ pain. The therapy was also focused solely on the orofacial region [18, 24] or on both—the neck and TMJ areas [11, 14, 26]. Even if the therapy was applied only to the neck, the study included patients with TMJ pain [11, 15]. In none of the existing studies, the effect of neck therapy on changes in TMJ has been assessed among patients with neck pain only and with no pain in the TMJ.

Some authors have suggested a relationship between neck pain and forward head posture [42–44]. Observations by other authors indicate that changes in head position may cause pain and dysfunctions of the cervical spine and masticatory system [45]. Cortese et al. [46] noted that forward head posture is a risk factor for TMDs. However, other authors reported that the occurrence of incorrect head and cervical spine posture is similar among people with and without temporomandibular joint dysfunctions [47, 48]. In our study, we found more cases of TMDs in participants with neck pain than in pain-free control participants. We also found improvement in TMJ function after the applied therapy. There are many contradictory reports on the relationship between head position and TMDs [49–51]. In some studies, it has been suggested that excessive forward head posture affects the head's centre of gravity position and, thus, the position of the mandible in the temporomandibular joint, gradually leading to dysfunction [52]. According to those authors, excessive head forward posture disrupts the mechanics of the cervical spine and may affect deep muscle tension [50, 52].

Forsberg et al. [53] investigated different lengths and tone of the neck muscles which may affect masticatory muscle activity. They suggested that limited cervical spine mobility could increase the degree of an already existing dysfunction. Therefore, physiotherapy focused on improving mobility in the cervical spine may also have positive effects on the proper functioning of the masticatory system. Furthermore, Calixtre et al. [26] have reported that mobilisation of the upper cervical region and craniocervical flexor training decrease orofacial pain and headaches occurring in women with TMD. Those findings correspond to those obtained in the current study, in which 76% of participants with neck pain demonstrated forward head posture. The implemented therapy caused corrected head posture and simultaneously decreased pain intensity. In our research, 76% of the experimental group demonstrated forward head posture compared to only 20% in the control group. After the rehabilitation programme, including education of correct posture with a neutral head position, the dysfunction decreased significantly (28%), reaching similar values as those obtained for the control group.

There have been some reports on the coexistence of neck pain and TMDs [18, 19, 54, 55]. In our study, more severe TMDs were found in patients with idiopathic neck pain than in controls. This maintains in agreement with other studies [20, 54], in which the association of TMDs and increased risk of neck pain is suggested [55, 56]. Bragatto et al. [14] verified an association between TMD and neck pain in computer office workers. They hypothesised that the percentage of subjects who were clinically diagnosed with TMD and pain in craniocervical structures would be higher in computer workers with self-reported neck pain and disability than computer workers who did not report chronic neck pain, as well as noncomputer workers. The results from their study showed that computer workers with neck pain have a twofold higher prevalence of TMD than those without pain.

There are also some studies in which it has been described how therapy of the craniofacial area may influence neck dysfunction. Walczyńska-Dragon and Baron [24] have found that applying TMD treatment (occlusal splint and self-control parafunctional habits) may improve neck range of motion and neck pain severity. They have also shown significant improvement in TMJ functioning and spinal movements, as well as a reduction in spinal pain after therapy with an occlusal splint [25].

In our study, it was observed that after rehabilitation, without any therapeutic intervention in TMJ, not only the pain in the cervical spine was less intense but also the level of masticatory system dysfunction significantly decreased. Our results could suggest that neck function improvement may additionally have a positive effect on the functioning of the masticatory system. Halmova et al. [15] have studied causality between the craniocervical dysfunction and myofascial pain in the head and neck as well as the clinical value of physiotherapy. The group of patients diagnosed with myofascial dysfunctional pain syndrome received standard therapy for TMJ, while in the second group, therapy was aimed at the cervical muscles only, and in the third group, the patients received complex rehabilitation. Unfortunately, the results of the therapy were evaluated only via the subjective visual analogue scale (VAS), without any objective measurement methods. However, they concluded that the therapy, which included exercises and manual and physical therapy, led to much differentiated, often-conflicting results, and those authors suggested that an individual approach to each patient is the most important. In their study, the authors have indicated the efficiency of relaxing and stretching exercises of cervical muscles in combination with widely used masticatory muscle therapy [15]. Furthermore, Maluf et al. [57] observed a decrease in pain intensity and tension of the neck and craniofacial muscles among patients with TMDs after stretching of the superficial neck and masticatory muscles. This phenomenon could be explained by the fact that the muscles of the masticatory system are synergistic or antagonistic to the neck muscles biomechanically as cervical spine flexors or extensors. Therefore, correct functioning of the entire cervical muscular system, with maintaining full mobility of the cervical spine, is a crucial element of healthy functioning of the masticatory system. Also, Marcos-Martín et al. [23], after biobehavioral therapy, observed improvement in self-reported disability, psychological factors, and craniocervical posture in patients with chronic myofascial TMD and neck pain.

In the current study, despite the lack of direct intervention in the craniofacial area, we have observed improvement in clinical condition of the TMJ. This observation showed a mutual correlation between the cervical spine and the masticatory system, which may be related to neurophysiologic connections between the cervical spine and temporomandibular area reported by a previous study [12]. Silveira et al. [19] evaluated the correlation among neck disability and TMJ dysfunction in subjects with TMD and concluded that high levels of neck disability are correlated with high levels of jaw disability. They underlined the importance of considering the neck structures when evaluating and treating patients with TMD.

It should be underlined that patients with neck pain but without pain in the TMJ may suffer from asymptomatic TMD. We have suggested that complex TMJ evaluation should always be included in the assessment and treatment of patients with idiopathic neck pain, even if they do not report orofacial pain. There are some studies in which a relationship is indicated between restricted range of motion in the cervical spine and neck pain [58]. Those observations are in agreement with our own results obtained in the experimental group, noting that decreased cervical extension and lateral flexion range of motion compared to the controls were noted, which improved after treatment with a simultaneous decrease in neck pain.

This study has some limitations which should be addressed. Due to the fact that we did not perform follow-up assessment after a longer period after completion of therapy, we do not know how long the effects of therapy last. Therefore, a study with a long-run follow-up period and covering patients with dysfunctions different from that of idiopathic neck pain is needed. Also, because of ethical reasons, we could not leave patients with neck pain without rehabilitation; therefore, the control group included the healthy age-matched subjects without idiopathic neck-pain. Thus, we can only consider them as a reference.

## 5. Conclusions

The rehabilitation programme lasting 3 weeks significantly decreased pain intensity and improved range of motion of the cervical spine and head posture as well as clinical condition of the TMJs in participants with idiopathic neck pain without reported TMJ pain. At baseline, the evaluated variables were significantly worse in the experimental group compared to control, but after the implementation of therapy, they reached values similar to those noted in the control group. Therefore, we may consider values in the control group to be normative, which can be a point of reference during therapy in subjects with idiopathic neck pain. The results of our research allow to suggest that idiopathic neck pain is associated with limited range of motion in the cervical spine, incorrect head posture, and dysfunction of TMJ. This study may indicate that therapy focusing only on the cervical region could improve clinical condition of TMJ in subjects with idiopathic neck pain but not reporting TMJ pain.

## Figures and Tables

**Figure 2:**
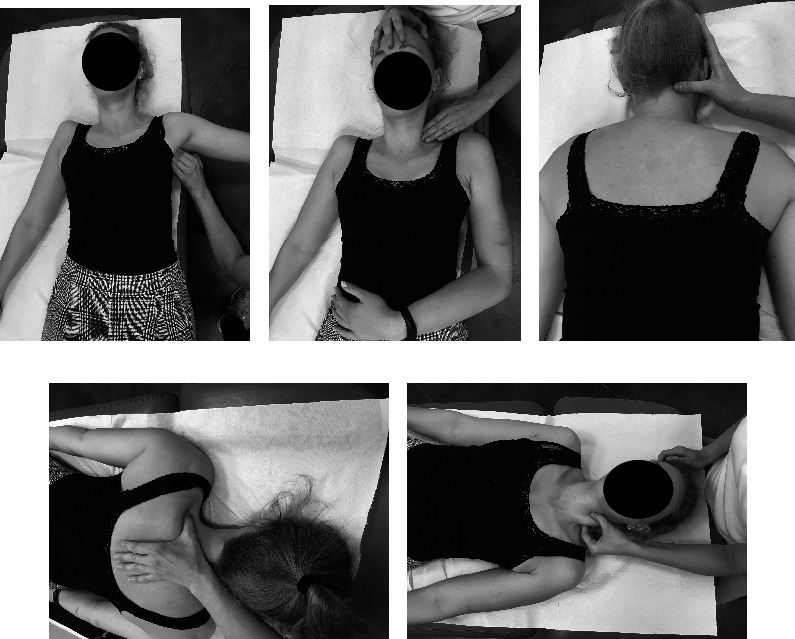
Trigger point therapy of the shoulder girdle and the neck: (a) m. pectoralis minor; (b) m. scalene; (c) m. cervical paraspinal; (d) m. trapezius; (e) m. sternocleidomastoid.

**Figure 3:**
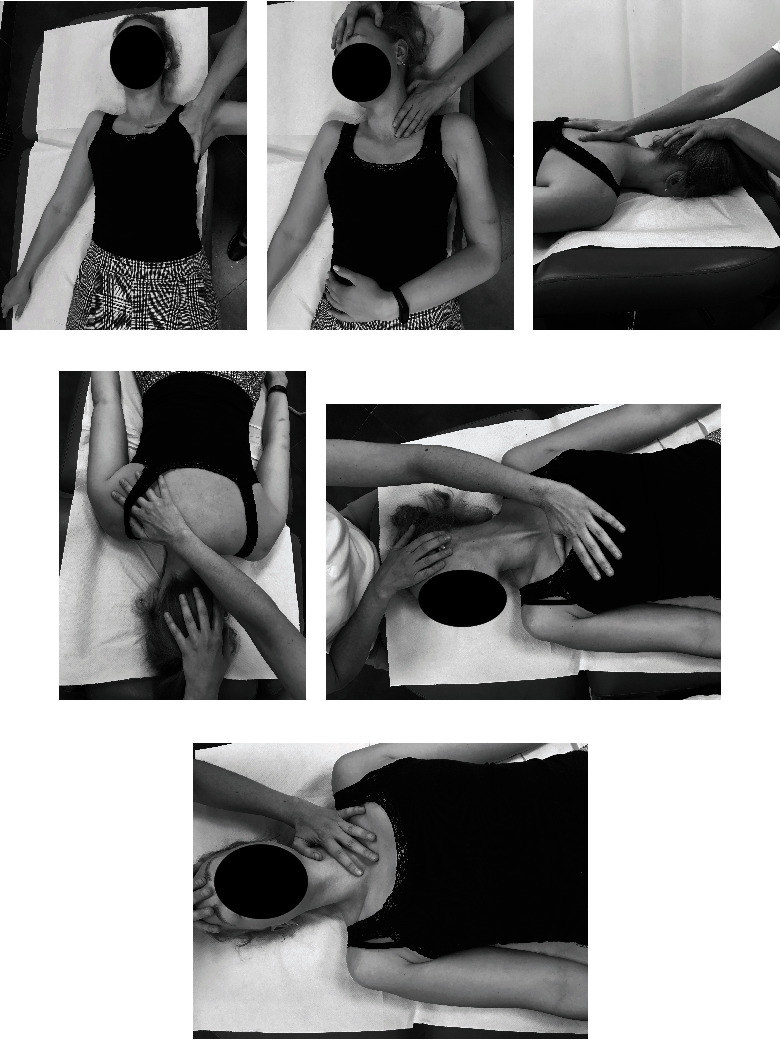
Myofascial release of the shoulder girdle and the neck: (a) m. pectoralis major; (b) m. scalene; (c, d) m. of the shoulder girdle; (e, f) m. sternocleidomastoid.

**Figure 4:**
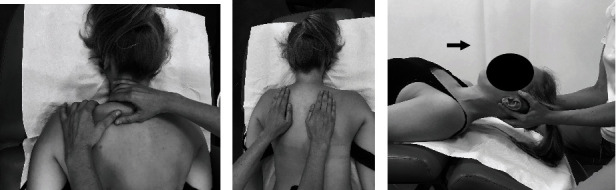
Classical massage of the shoulder girdle and the neck (a, b) and manual cervical traction (c).

**Figure 5:**
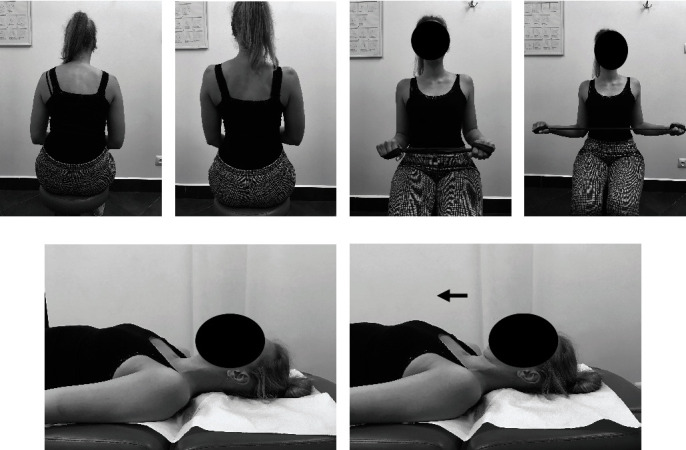
Active exercise of the shoulder girdle ((a) starting position; (b) activity; (c) progression, starting position; (d) progression, activity) and the neck ((e) starting position; (f) activity).

**Figure 6:**
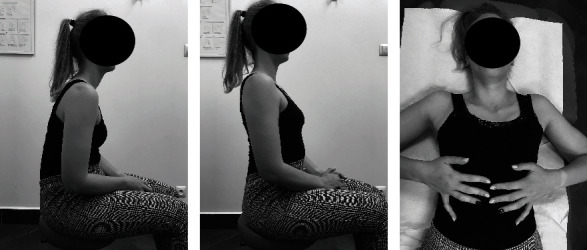
Body posture correction exercises ((a) starting position; (b) activity) and respiratory reeducation (c).

**Table 1:** Patient characteristics.

	Experimental group	Control group
Number of subjects (*n*)	25	35
Sex	21 women, 4 men	29 women, 6 men
Age (years)	27-57 (38.5 ± 8.52)	27-47 (35.1 ± 5.65)
Body mass (kg)	62 ± 11.9	64.3 ± 14.1
Body height (cm)	164.6 ± 6.3	166.2 ± 5.3
Physical activity level	Recreational	Recreational
Functionality	Professionally active	Professionally active

**Table 2:** Head posture in the sagittal plane at baseline and after therapy.

Outcome measure		Experimental group	*p* ^#^	ES^#^	Control group	*p* ^#^	ES^#^	*p* ^∗^
Distance in habitual head posture (cm)	Baseline	13.1 ± 1.7	0.0001	0.43	12.4 ± 1.6	0.96	0.06	0.10
	Post	12.4 ± 1.5			12.3 ± 1.4			0.98
Distance in corrected head posture (cm)	Baseline	11.2 ± 1.9	0.009	0.36	11.8 ± 1.9	0.56	0.03	0.56
	Post	10.5 ± 1.9			11.8 ± 1.7			0.006

^#^ *p*: *p* value between baseline and posttherapy within each group (time main effect). ^∗^*p*: *p* value between study groups (group main effect). ^#^ES: effect size (Cohen *d*) within each group. Values are expressed as the mean ± SD.

**Table 3:** Range of motion in the cervical spine at baseline and after therapy.

Outcome measure		Experimental group	*p* ^#^	ES^#^	Control group	*p* ^#^	ES^#^	*p* ^∗^
Flexion in upper segments (cm)	Baseline	5.5 ± 1.9	0.97	0.04	5.2 ± 1.9	0.93	0.05	0.85
	Post	5.5 ± 1.9			5.1 ± 1.8			0.87
Flexion in lower segments (cm)	Baseline	9.9 ± 2.2	0.84	0.19	10.2 ± 2.2	0.92	0.05	0.79
	Post	9.5 ± 1.8			10.1 ± 1.6			0.81
Extension in upper segments (cm)	Baseline	5.3 ± 1.5	0.001	0.58	6.5 ± 1.8	0.96	0.04	0.01
	Post	6.3 ± 1.9			6.5 ± 1.8			0.94
Extension in lower segments (cm)	Baseline	6.8 ± 1.9	0.0001	0.66	7.5 ± 2.0	0.94	0.05	0.53
	Post	8.0 ± 1.7			7.6 ± 1.9			0.84
Rotations to the right (cm)	Baseline	10.7 ± 2.2	0.0001	0.62	11.4 ± 2.5	0.99	0.04	0.83
	Post	11.9 ± 1.6			11.3 ± 2.5			0.82
Rotations to the left (cm)	Baseline	11.5 ± 2.2	0.067	0.23	10.9 ± 2.2	0.99	0.04	0.74
	Post	12.0 ± 2.1			10.8 ± 2.1			0.04
Lateral flexion to the right (cm)	Baseline	5.9 ± 1.5	0.0001	0.83	6.9 ± 1.9	0.98	0.02	0.02
	Post	7.2 ± 1.6			6.9 ± 2.0			0.90
Lateral flexion to the left (cm)	Baseline	5.6 ± 1.7	0.0001	0.75	6.6 ± 1.7	0.97	0.04	0.02
	Post	7.0 ± 2.0			6.6 ± 1.8			0.85

^#^ *p*: *p* value between baseline and posttherapy within each group (time main effect). ^∗^*p*: *p* value between study groups (group main effect). ^#^ES: effect size (Cohen *d*) within each group. Values are expressed as the mean ± SD.

## Data Availability

All data are included within the manuscript.
